# Identity Formation and Developing Meaningful Social Relationships: The Role of the Polish Catholic Community for Polish Young People Migrating to Sweden

**DOI:** 10.3389/fsoc.2021.660638

**Published:** 2021-05-07

**Authors:** Oksana Shmulyar Gréen, Charlotte Melander, Ingrid Höjer

**Affiliations:** ^1^Department of Sociology and Work Science, University of Gothenburg, Gothenburg, Sweden; ^2^Department of Social Work, University of Gothenburg, Gothenburg, Sweden

**Keywords:** young people, mobility within the EU, religion, identity, relationships, Polish Catholic community, Sweden

## Abstract

This article draws from a broader research project *Transnational childhoods*, illuminating the agency and experiences of children and young people migrating from Poland and Romania to Sweden under the age of 18. Focusing on young people born in Poland and having social relationships post-migration as central theoretical component, the article explores the role that the Polish Catholic community in Sweden plays in the lives of young Polish migrants. It does so by grounding the analysis on 23 qualitative interviews, combined with network maps and life-lines, produced by the young Polish participants. The study identifies three important dimensions in the role of the Polish Catholic community. These are comprised of the community's role for young Poles' spiritual development and religious identity, for building new friendships and making sense of common migration and religious experiences, and guidance by specifically Polish Catholic priests in the young migrants' family relationships and in future life projects. The article concludes that while practicing religion and building significant social relationships within the Polish congregations the young migrants shape feelings of belonging and inclusion, however primarily within the limits of their own ethnic community. Further research is needed on the wider implications of primarily mono-ethnic relational practices for the young Poles' lives within the increasingly ethnically heterogeneous Swedish society.

## Introduction

The issue that underpins this article is the integral role that the Polish Catholic community in Sweden comes to play in facilitating young Polish migrants' identity construction, and in developing caring social relationships post-migration. Our interest in analyzing religious and spiritual components in young migrants' everyday lives has largely been stimulated by the empirical data emerging from a broader research project: *Transnational childhoods*.^1^ This project follows on from our previous research on Polish and Romanian transnational families settling in Sweden following the accession of these countries to the EU (Melander and Shmulyar Gréen, 2018; Shmulyar Gréen and Melander, 2018; Melander et al., 2020). The overall objective of the project is to illuminate the agency of children and young people and their experiences of reshaping caring social relationships after migrating to Sweden under the age of eighteen. Project participants' own migration is, as a rule, a result of their parents' moving to Sweden for work, where Poland and Romania represent the two main countries of birth among recent arrivals among Central and Eastern European (CEE) EU citizens (Statistics Sweden (SCB), 2019).

In this article, we focus on young people born in Poland. Without sampling for young migrants interested in religion or asking overtly about their religious beliefs, during the course of the interviews we became increasingly aware of the fact that, in contrast to the Romanian participants, almost all of the Polish participants described themselves as being Catholics. Young Poles' formal affiliation with and the density of their activities in the Polish Catholic Church in Sweden varies both over time and from person to person. However, a common pattern that emerged from the empirical data was that the local Polish Catholic community in Gothenburg, as well as other Catholic and missionary congregations, were repeatedly mentioned by the young Poles as meaningful sites for developing deep social relationships post-migration. In particular, for several participants the Polish Catholic community, with its many resources, various social activities and some specific religious actors within it, came to play an essential role during the initial phase of settling in Sweden, coinciding for many of them with preparations for their Catholic confirmation. For some participants, the social relationships built up during that time remain their primary personal and mentoring relationships to this day. In this sense, the Polish Catholic community stands out as an important social place and space (Haikkola, 2011), through which young people actively build and transform their sense of belonging and social recognition in a new country.

Having observed these patterns, the article proposes to further examine and theorize about them, especially in light of the somewhat contested position that the Catholic faith, and religion in general, seems to occupy among young people in Sweden. One of the few existing studies on the Polish Catholics in Sweden clearly indicates that young people are “simply absent” in Polish Catholic services (Drigo, 2017, p. 47). Other studies of Catholic young people in Sweden, many of whom are of immigrant background, have noted that young people identifying themselves as Catholics often feel questioned by their Swedish-born peers, especially in relation to the morality rules of the Catholic religion (Salminen-Karlsson, 2005; Lundberg, 2010). Being expected to defend these rules, young Catholics face a lot of pressure, leading some of them to either avoid speaking up about their religiosity or dissociating themselves from the Catholic Church altogether. Moreover, as Sjöborg (2015), von Brömssen (2016), and others observe, among young Swedes, religion “is constructed as something which is distant in time or place, which is not associated with modernity, progress, and science” (Sjöborg, 2015, p. 126). In a sense, when talking to young Poles whose upbringing to a large extent took place in Sweden, we anticipated a certain congruity with the views of their peers. The Church of Sweden's official estimates also attest to the fading bonds to the church and religiosity among ordinary Swedes, as both formal membership and religious practices have drastically declined during the last 30 years (The Church of Sweden). In line with this, some researchers argue that “non-religious attachment to a church is the final remnant of religion in Sweden” (Kasselstrand, 2015, p. 291).

Having said that, young Poles' increased or strengthened identification as Catholics post-migration deserves further attention. With social relationships post-migration as a focal point, the aim of this article is to explore the role that the Polish Catholic community in Sweden plays in the lives of young Polish migrants in their identity construction and in developing meaningful and caring social relationships. The analysis is developed in relation to three important dimensions: young Poles' spiritual development and religious identity, building new friendships and making sense of common migration and religious experiences, and guidance by specifically Polish Catholic priests in the young migrants' family relationships and in decision-making about their futures.

## The Catholic Church and the Polish Catholic Mission in Sweden

To provide an understanding of the specific context of the Polish Catholic Church and community in Sweden, it is important to outline its position in a society with largely secular dispositions. Once the state church of Sweden, the Catholic Church is now a minority church, accounting for up to 125,000 members in 44 parishes all over the country (The Catholic Church in Sweden). In contrast, the Church of Sweden, despite a substantial decline in membership over the years, has around 56% of Swedes still declaring themselves to be members, and is “where three out of four funerals take place, and where close to half of Swedish newborns are baptized” (Kasselstrand, 2015, p. 278).

The rise and fall of Catholicism in Sweden has a contentious history and it is only from the mid-1970s, when Sweden adopted a principle of religious freedom in its Constitution, that the Catholic Church gained a growing acceptance in the society and consequently a rising number of members (Drigo, 2017). Migration and migrants' transnational ties have played an integral role in increasing the linguistic and cultural diversity of the Catholic Church in Sweden (Lundberg, 2010; Erdal, 2016). According to the official webpage, in Sweden, the Catholic Church includes 17 dioceses conducting masses in nine different languages, besides Swedish (The Catholic Church in Sweden). The majority of members of the Catholic Church in Sweden are migrants themselves and come from Poland, Croatia, and different countries of Latin America (Lundberg, 2010; Drigo, 2017).

During the last two decades, a common trend observed in Sweden, Norway, and the UK is that since the 2004 EU enlargement, Polish-speaking Catholics as well as Polish-speaking priests have contributed to a significant demographic shift among Catholics in Sweden (Trzebiatowska, 2010; Erdal, 2016; Drigo, 2017). Delmi (2016) estimates that among nationals from the 28 EU countries residing in Sweden in 2016, 89,000 were born in Poland. To these should be added ~10,300 children and young people born in Poland and residing in Sweden together with their families in 2018 (Statistics Sweden (SCB), 2019). Arguably, not all Polish migrants are Catholics, and not all Polish Catholics are active churchgoers. Even so, the substantial numbers of Poles arriving and settling in Sweden since the enlargement is one of the main reasons for a growing number of Catholic congregations where Polish is the main liturgical language. Due to the specific nature of the post-enlargement migration, the Catholic Church in Sweden, similar to other migrants' destinations within the EU, often functions “as ‘a harbor of hope and worship' for many economically vulnerable migrants” (Trzebiatowska, 2010, p. 1059).

It is important to note that, similar to the UK, migration from Poland to Sweden in significant numbers started already after WWII. Among the post-war refugees, there were quite a few Polish priests, which led to the establishment of the Polish Catholic mission in Sweden in 1953 (Drigo, 2017). The recruitment of Catholic priests from Poland to Sweden intensified over the years since. As Salminen-Karlsson (2005) observes, the Catholic Church became almost dependent on the clergy coming from Poland due to many vacancies in the Swedish parishes. Thus, unlike in other Nordic countries, where the Catholic parishes seemed to be unprepared to serve the newly arrived migrants from Poland (Erdal, 2016), in Sweden the Polish Catholic community evolved gradually and was better equipped to meet their needs.

The Catholic parish in Gothenburg, which most of our participants mention in their stories, was founded in the mid-1980s and is run by the priests and brothers of the Capuchin Order, one of the branches of the Franciscan first orders. The Capuchin Brotherhood was established in Sweden in 2003 and many of its brothers arrived in the aftermath of the Polish accession to the EU. Currently, the local parish in Gothenburg runs eight groups in religious education for children between 7 and 16 years old; altogether 50 young people (The Polish Mission in Gothenburg). There are also youth organizations that bring together young Catholics in Sweden. Several of our participants mentioned the Swedish Young Catholics (SUK). SUK has been registered as the National Youth organization of the Catholic Church since 1975 and currently engages around 3,000 young Catholics in Sweden between 6 and 28 years old in six regional branches (Salminen-Karlsson, 2005, SUK). Another organization, whose activities are often attended by participants in our study, is the Polish Young Catholics (PUKIS), established in 1989. It currently operates in three regional branches bringing together young Poles for Catholic camps, pilgrimages, and music festivals.

What emerges from the discussion above is that young Poles practicing Catholicism in Sweden are part of a religious minority within a diverse minority Church, but also an ethnic minority within multicultural Swedish society, both positions being novel and challenging for the young people. This presents an important backdrop against which we analyze the narratives of the young Poles.

## Perspectives on Young People's Mobility Within the Eu, Religion and Identity Construction

Research on young people mobility within intra-EU family migration has largely been focused on young people's familial and educational adaptations in their host societies (e.g., D'Angelo and Ryan, 2011; Sime and Fox, 2015a,b; Moskal and Tyrrell, 2016; Assmuth et al., 2018; Pustulka and Trabka, 2019). Family and schooling are indeed two of the most central domains in which, due to migration, young people experience a great deal of transition and adaptation. Rather than seeing children's own mobility as a unique and isolated event resulting in a number of normative transitions, we take a long-term view by adopting a life-course perspective (Wingens et al., 2011). What becomes apparent, especially through the lens of the linked-lives principle, is that the children's mobility is inherently embedded in their parents' initial migration, the children's own migration, and reunification with parents in Sweden. Furthermore, children's mobility is often marked by ambivalent feelings, some of which are excitement, expectation and longing, with others being loss and anxiety (Sime and Fox, 2015a; Melander and Shmulyar Gréen, 2018).

To provide further insights into these processes, we focus on the relational nature of children's and young people's mobility, especially during their post-migration socialization in translocal spaces (Assmuth et al., 2018). We argue that having a capacity to develop meaningful social ties is crucial for children and young people in order to access resources and manage the transitions that migration implies. As Haikkola (2011) demonstrates, a child-centered approach is required in order to examine how migrant children and young people shape and transform social relationships that they consider important, and how these relationships impact on their identities. Seen from this angle other social places and spaces such as, for instance, faith communities, may emerge as essential for migrant children's and young people's identity constructions.

Migrating at a younger age and growing older in a new country sets in motion “the reflective project of self” (Holland et al., 2007, p. 101), involving multiple identity constructions composed of “adult identity, ethnic identity and children's self-identity that is independent from their parents” (Holland et al., 2007). Accordingly, one can expect that in moving to Sweden, young Poles (as well as their parents) experience a profound shift from a culturally and religiously homogeneous and largely mono-ethnic society to a Swedish multicultural society “dominated by a secularist discourse” (von Brömssen, 2016, p. 122; Drigo, 2017). Coming of age in a rather contrasting social environment may have far-reaching implications for migrant young people's identity-seeking processes, being closely intertwined with the creation of new, meaningful relationships post-migration in the host country, as well as a struggle to maintain ties with the country of their birth.

In order to highlight how children navigate these processes, we evoke the concept of the “care worlds of children” developed by Lynch et al. (2009) and McGovern and Devine (2016). This notion links together the relationships of love, care and solidarity, which as Lynch et al. (2009, p. 38) assert, constitute an affective domain in children's lives, vital in order “to establish a basic sense of importance, value and belonging, a sense of being appreciated, wanted and cared about.” Furthermore, Lynch et al. (2009) maintain that the intimate ties between parents and children are described as the prototypical relationship based on love, usually with a high level of interdependence and emotional engagement. Care relationships are situated within the circle of ties to relatives, friends, or teachers usually playing a secondary role in the order of engagement. Finally, relationships of solidarity constitute collective forms of commitment, including the feelings of reciprocity, encouragement, and mutual affirmation that children can establish, for instance, within their social activities in associations, sport clubs, church groups, etc. Paying closer attention to the migrant children's affective practices revealed that the Polish Catholic community and the specific actors within it could be found across all three domains of the young Poles' caring relationships.

It is important to note that as part of the Catholic faith, every believer has to develop their own relationship with God (Salminen-Karlsson, 2005). In their quest to know God, young people often rely on guidance from their parents, but also, as we show below, on the religious leaders within the Catholic Church, including priests and monks. In this sense, the religious leaders become both religious educators and carers for the young people's spirituality; for instance through the practice of confession and guidance. To understand this relationship, we rely on Reynaert's (2014) conceptualization of Foucault's notion of pastoral power, performed through both care and control. As Reynaert (2014, p. 185) explains, while nurturing children's spirituality, one has to be aware of “the hidden forms of power,” even if pastoral power is not necessarily a hierarchical relationship. In relation to the phenomena we studied, pastoral power illuminates young people's willingness to trust that the religious leader's care and guidance is done in young people's best interests (Reynaert, 2014). At the same time, the fact that the young Poles in our study migrate and grow up in a largely secular Swedish society may produce tensions in the role that the Polish Catholic priests and monks are supposed to perform in relation to their laity. On the one hand, they might be regarded by the young people as attentive listening adults, “encouraging wondering and questioning” (Reynaert, 2014, p. 181) in their search for spirituality and universal Catholic values. On the other hand, as observed by Trzebiatowska (2010), there are some limits on the Polish clergy's habitus, especially in relation to migrant faith communities in secular contexts. Trzebiatowska (2010, p. 1064) further explains: “Polish clergy are concerned that Polish children ‘brought up in a permissive and secular society will lose their faith and make wrong life choices.'”

Going further, this article aims to shed some light on how migrant children develop meaningful social relationships within the spiritual and religious realms, expanding beyond family and schooling, and yet deeply embedded in them. As von Brömssen (2016) observes, religion and young people, as a field of study, is now on the rise in Europe as a whole. At the same time, it is acknowledged that religion has become a highly politicized subject, not least in relation to its role in migration, multiculturalism and integration processes in Europe (Lundberg, 2010; Trzebiatowska, 2010; Sjöborg, 2015; Erdal, 2016). Arguably, as several researchers underline, Catholicism has a central position in Polish identity. However, it is not uncommon that the link between the “national” and the “religious” Polish identities is taken for granted and seldom gets further examination in migration studies (Salminen-Karlsson, 2005; Trzebiatowska, 2010; Drigo, 2017).

Our perspective will be to focus on the everyday practices and activities, combining religious and non-religious expressions, which young Poles identify as meaningful when they participate in the Polish Catholic community in Sweden. This means that instead of examining formal religious affiliation, organizational participation, or the theological doctrine of the Catholic Church, the article focuses on how and why young Poles assign meaning to the Catholic community and its specific religious leaders, and the relationships they develop within it. This line of inquiry is enabled by the conceptual lenses of lived religion and spirituality. The perspective of lived religion brings to the fore the actual practices and relationships by which young people “make up their personal religious experience and expression” (McGuire, 2008, p. 2). The logic of religion-as-lived, as McGuire (McGuire, 2008) asserts, is not necessarily coherent with the hegemonic definitions of what religion is supposed to be. As McGuire (2008, p. 12) explains: “Rather, it requires a practical coherence: It needs to make sense in one's everyday life, and it needs to be effective, to “work,” in the sense of accomplishing some desired end (such as healing, improving one's relationship with a loved one, or harvesting enough food to last the winter).” Explored in this light, religion as a lived experience is closely intertwined with the notion of spirituality which, while it might be religious in its nature, in broader terms stands for “a search for meaning, in which children attempt to make sense of life and of the world around them” (Adams, 2009, p. 115).

The article highlights that it is in and through the significant relationships forged within their care worlds that young people can make sense of and manage their experiences, as they ponder questions such as: Who am I in a new country? Who cares about me? How can I be a good person in relation to my parents, friends, wider kin “here and there?” How do I feel about the changes caused by migration? Even if some of these questions might be an essential part of young people's coming of age in general, we argue that migration experiences accentuate the search for the spiritual as well as a social space to believe in and belong to, due to the emergence of both age-related and migration-related transitions.

## Methods

Following up the voices of children in post-accession European mobile families in Sweden is a novel and still emerging research field, in which we are making the first exploratory steps. This study was not intended to be a representative one. Rather our intention was to collect rich qualitative data to gain -insights into the complex processes of building social relationships through the young people's migration processes. In the research project, all in all we met 17 participants from Poland and Romania and conducted in-depth interviews in two steps, amounting to 34 interviews, ~68 h of recorded conversations, and more than 1,700 pages of transcribed text. Focusing on Polish participants alone, the article is drawn from 23 qualitative interviews, life-lines, network maps, and other visual material from nine female and two male Polish-born young people. We interviewed most of them on two occasions, and some on one or three occasions from May 2019 to December 2020. Having learned from our previous experience of doing research with Polish and Romanian transnational families in Sweden (e.g., Shmulyar Gréen and Melander, 2018), the recruitment of interviewees took place through multiple channels, including mother-tongue language courses, Facebook posts, visits to Polish church services, contacting gate-keepers within the Polish community in Gothenburg and some snowballing. On all occasions, we presented the aim of the research and provided examples of the interview questions, using the short format of recruitment cards but also comprehensive information letters including all aspects of the research process in Swedish or, if preferred, translated into Polish. The outcome of the recruitment process showed that having a gate-keeper, a young Polish-born university student, was the most effective way of gaining trust and being put in contact with new participants through snowballing. The criteria for selection were young Poles who had arrived in Sweden before turning 18 to join one or both parents, and they were supposed to live in the country for a least 2 years (see Table 1 below).

**Table 1:** Polish-born research participants.

**Participants' pseudonym**	**Age at the time of interview**	**Age on arrival in Sweden**	**Recruitment channels**	**Spiritual and religious activities within the PCC in Sweden mentioned by the informants**
Monica	23	11	Gate-keeper within the Polish community	Regular confessions and Sunday masses, taking part in catechesis, yearly meetings with the priest.
Antoni	25	18	Gate-keeper within the Polish community	Polish Catholic youth camps, regular confessions, hang around in the church together with other young Polish Catholics.
Matilda	21	9	Gate-keeper within the Polish community	Taking part in catechesis, occasional confessions, former leadership role within the Polish Catholic youth community, Polish Catholic youth camps, regular Sunday masses and socializing at the church coffee gathering with friends and the priests, weekly meetings with the Polish young Catholic group.
Faustyna Maj	23	15	Gate-keeper within the Polish community	Taking part in catechesis, Polish Catholic camps, everyday praying, regular confessions, active in the Swedish Catholic community, occasional taking part in Swedish Young Catholics group.
Jan	21	13	Snowballing	Taking part in catechesis, Polish Catholic camps, regular confessions, hang around in the church together with the Polish young Catholic group.
Märta	17	10	Mother-tongue language courses	Taking part in catechesis every second week at the time of the interview, Polish Catholic camps.
Anna	16	12	Snowballing	Taking part in catechesis, Polish Catholic camps, receiving guidance and emotional support from her confirmation teaching priest.
Amanda	28	14	Snowballing	Taking part in catechesis, leadership role in the Polish Young Catholic group, occasional taking part in Swedish Young Catholics group, regular confession, everyday praying.
Antonina	29	16	Facebook posts within the Polish community	Play an active role as a member in another non-Catholic Christian congregation.
Vera	18	6	Snowballing	Taking part in catechesis, Polish Catholic camps for young people, sporadic confessions.
Wanda	19	7	Snowballing	Taking part in catechesis, occasional confessions, Polish Catholic camps for young people.

Doing research in the middle of the pandemic has required significant adjustments to our methodological strategies. For instance, physical access to schools, ethnic group associations, and other places where we could meet young people or their parents face-to-face was instantly forbidden due to the restrictions. Thus, out of 23 conducted interviews, five had to be carried out *via* Zoom, which expanded the physical locations of our interviews from the city of Gothenburg and its close vicinity to, on a few occasions, other cities in Sweden. Recruiting young people willing to participate in the research has been both a rewarding and challenging process. For instance, we had to renounce our initial intention to interview children of younger age (below 15 years old). This was partly because a few parents, whose consent was necessary for us to interview children younger than 15 years old, were hesitant to allow us to talk with their children on issues of family and migration. At times young people older than 15 years have themselves refused to participate because they were still struggling with the difficulties caused by migration, and did not feel ready to share these stories. Those who chose to take an active part in the study instead tended to justify their participation by wanting their story to be heard and listened to. Some had a hope that it might lead to a change in the situation for other migrant children who were struggling with social exclusion and bullying in school. They wanted adults, especially teachers and parents, to know how important it is to listen and pay attention to migrant children's needs, and to actively work against oppressive interactions among peers.

All 11 Polish participants came to Sweden with their families post-2004, usually joining their fathers who came to work in the country after the Polish accession to the EU. At the time of interviewing, their ages ranged between 16 and 29 years old; some were enrolled in high school, while others were studying at university or working. Contrary to our initial intention to engage more recent arrivals in our study, the interviews have turned out to be more retrospective in character, as some young Poles had arrived in Sweden 4 years ago, and others had lived in the country for 12 years. All interviews were conducted in Swedish and transcribed verbatim. The participants were mainly bilingual and used Polish more or less actively in their everyday lives.

The project was granted Ethical approval by the Swedish Ethical Review Authority [Reg. no.: 2019-02504]. Following the ethical guidelines, we secured the participants' anonymity and integrity by asking them to choose a fictitious name and by carefully concealing other personal data to avoid easy identification.

## Analytical Strategies

Inspired by the methodologies studying children as active agents in migration (Haikkola, 2011; Pirskanen et al., 2015; Sime and Fox, 2015a,b), we combined various qualitative techniques in order to map out young people's relationships to social places and spaces that they value the most, both retrospectively and in the present. All these data sets for each participant were initially sorted and analyzed using the life-course perspective. The life-course perspective (Wingens et al., 2011) reclaims the central importance of time, which in relation to children's mobility and migration reveals continuities and turns in their lives, linked to their own experiences but also to societal opportunities and constraints.

More specifically, when going through the life-lines produced by our participants, we were able to identify specific life events related to building new significant relationships, but also to discern when young people referred to faith and guidance within the Polish Catholic community in Sweden as decisive in managing their translocal adaptations. In a similar vein, when asked to map out the networks of people important to them in relation to mutual care and support, young participants in the study usually describe a complex of various social units. They identified particular individuals within their close and extended family, circle of friends, schooling and churches as those by whom the participants felt loved and appreciated, supported and listened to. Analyzing the network maps helped us to shed light on the importance of the specific relationships, connections, and social contexts through which children develop significant relationships, attachments, and feelings of belonging translocally. In doing so, we could identify the Polish Catholic community and the specific actors within it as among the most important personal networks identified by the young Poles.

We undertook a more detailed analysis of the community and its activities, using the grounded theory approach by Charmaz (2015). According to Charmaz (2015), it is helpful to apply an analytical approach during the interview process, which definitely directed our attention toward the Polish Catholic community as an important social arena for our participants. By raising questions about what was actually happening in the data and what people were doing within this social arena at the interview stage, grounded theory enabled a process of theorizing to emerge through coding, memo writing, and theoretical sampling, linking the data and the coding in a co-constructed process.

## Findings

### Young Poles' Spiritual Development and Religious Identity

As Adams (2009) points out, there is a paradox in many Western societies when it comes to hearing the voices of children on everyday matters. On the one hand, children are asked about their opinions and views on education, on consumption preferences, on living arrangements in cases of divorce, and the like. However, as Adams (2009, p. 117) underlines “when the spiritual aspect of a child's life is considered, many children remain silent.” What helped us to notice young migrants' spiritual quests was to carefully listen and recognize what they found to be most important in the cultural, social and emotional processes that were shaping their everyday relationships post-migration. We began all interviews with open-ended questions such as: *How would you present yourself? What makes you happy or sad? What is important in your life right now?* As the interviewing progressed, it became obvious that most of the participants mentioned family and friends as being of key importance in their lives. Of almost equal importance, quite a few mentioned Catholic priests and monks, and some even talked about “God” and “faith” as if they were humans who guided the young person in their everyday lives. Probing these answers further, the interviewees clearly indicated that leaving their home country as children set in motion a lot of fundamental questions, caused by multiple difficulties in adapting to their new environment, but also discoveries while rebuilding relationships in a new country.

The young Poles we have interviewed so far arrived in Sweden in their early school years or early and later teens. Some of them were quite keen on the idea of moving, curious about a new country, wanting to learn a new language, and finally being able to live “together as a family” after a number of years of living apart from their fathers. An overwhelming impression surfacing from the interviews was, however, a feeling of estrangement. Monica (23, 11 years old on arrival) remembers that in her family she was the one who was especially against leaving Poland. She spoke of being angry that she didn't have a choice to stay and this anger had, in the beginning, a significant effect on her perception of who she was as a person and on her capacity to deal with new relationships in Sweden:

*[I had a feeling of] missing, loss of people and identity. It was not okay to be the person I was. Others distanced from me, [they] were afraid of me. We went back to Poland 3–4 times a year – I realized that I was not the same person – did not fit in there and not in Sweden either*.

What Monica's statement portrays is a loss of “being recognized” and “accepted as she is” among both peers in Sweden but also when visiting her home country. This sudden uncertainty, displacement, and in-betweenness, which Monica puts into words, has been observed in other studies on European migrant children and young people in various destinations (Sime and Fox, 2015a; Slany and Strzemecka, 2016; Tyrell et al., 2019). Adding to these important debates, this article brings to the fore how young migrants cope with this uncertainty and find their place in a new country. Despite their young age, a few of our participants admit that they had to deal with the feelings of estrangement and loss on their own, especially straight after their arrival in Sweden. Matilda (21, 9 years old on arrival), explains:

*In the beginning, when we came to Sweden, I felt I did not really recognize myself here. I complained to my father once in a while, but my father, he is pretty […] cold-hearted, so he would have thought I exaggerated if I said that I did not have any friends and wanted to go back to Poland. So I let it be, I did not complain anymore, I just continued with my life as it was*.

Similar to the findings in Sime and Fox (2015a, p. 383), for Monica, Matilda and other young Poles in the study, “Life post-migration was not always as the children had imagined it.” While parents had to work long hours to make a living in a new country, and with extended family members being far away, the participants had to learn to rely on themselves as a strategy to cope with the feelings of loneliness and longing. A few of them recall that after school they spent a lot of time alone in their homes, reading literature and poetry in Polish, or listening to their favorite music. Some girls described how they “locked themselves in their own rooms,” watching movies or playing computer games. For some young people, these were comforting and meaningful activities, illustrating young migrants' agency while adapting to a new situation. For others, the reluctance to socialize could be an indication of a quest for recognition and inclusion in other social domains, beyond their families and school.

Parents, and especially mothers, were usually aware of their children's feelings of insecurity. Being themselves newcomers to Swedish society, mothers were the first to actively search for Catholic masses in Polish and guidance on how to assist their children to join youth groups and preparing for their first communion or confirmation. Preserving religious identity as Catholics is quite important for many Polish migrants abroad (Trzebiatowska, 2010; Erdal, 2016). In Sweden, as Drigo (2017, p. 51) indicates, some Polish parents are particularly concerned with the fact that “the Swedish lifestyle influences negatively the religious life of Polish young Catholics” who in their view are more malleable and may lose their religious practices. Faustyna Maj (23, 15 years old on arrival), despite being positive about moving to Sweden, felt quite lonely during the first year after migration. She remembers her initial unwillingness to join the church's activities with some irony in her voice:

*It was my mother who forced me to attend the Christian religion classes (catechesis) for Polish children. [I: Did she really force you?] You could say, she strongly encouraged me. I wanted to stop attending church when I came here. It felt so pointless we were not very religious after all*.

As this statement indicates, Faustyna Maj's mother exercised pastoral power in Reynaert's terms (Reynaert, 2014) both as a caregiver and as a parent concerned about her child's spiritual life. At the same time, seen through the prism of care and love relationships (McGovern and Devine, 2016), the mother might have expressed her affection and empathy in the hope that within the Catholic community her daughter would feel accepted and welcomed.

In line with Lundberg (2010, p. 157), migrant parents tend to transmit the resources and the structures for religious education to their children because “religion is a tool for parents to reproduce their own culture in the children, and congregations make up a place for intra-group dynamics, in relation to generations and the sexes.” This tendency, and especially the mother's role in passing on the religious tradition to their children, is well-documented in other research on migrants' religious identities (Levitt et al., 2017).

However, as Faustyna Maj indicates in the quote above, even though they come from a country where the Catholic religion has a central role in many aspects of social life, younger generations do not automatically become active church-going Catholics in Sweden (see also Drigo, 2017). Most of the participants we interviewed received their first communion back in Poland before they migrated to Sweden. Wanda (19, 7 years old on arrival) was one of those who received her first communion in Sweden, but when her confirmation was approaching, she was a bit more hesitant.

*Me and my friends were a bit annoyed with one priest, who insisted on us going to the camp organized by the Polish Catholic Church. He said: You should go at least once in order to receive your confirmation. […] Ah, then people just went to the camp. Because within the Catholic Church, you know, everybody wants to be a part of the sacrament of confirmation, otherwise it will be difficult later if you choose to marry in the church*.

Somewhat surprisingly, from our point of view, was that Wanda and quite a few others admitted that after joining the catechesis classes in the Polish church in Sweden they seemed to discover God and practice their faith anew. When reasoning about the difference between being a Catholic believer in Sweden as compared to Poland, Amanda (28, 14 years old on arrival) clarifies:

*In Poland people go to church because they follow tradition. For me, faith is my life. [I: how come this difference?] I think it is because I had to […] how shall I put it? Fight for my faith. We have so many different cultures here in Sweden. It is not always easy to be a believing Catholic here. Especially these days, when priests are not always [good]*.

Amanda's and Wanda's reasonings disclose several important points. To begin with, for them and some other participants in the study, following the religious tradition of their parents and kin back in Poland is no longer an obligation. Rather, as Drigo (2017) and Salminen-Karlsson (2005) highlight, for many young Poles growing up in Sweden, religion becomes a marker of personal identity and individuality, and in some sense a matter of personal choice to be spiritual. It is also possible to argue that Amanda, Wanda, and other young Poles became suddenly aware of their dual minority status in Sweden, both as migrants and as Catholics. Thus, “choosing to believe” is also a strategy to break loose from being perceived as different. As participants' life lines clearly indicate, joining the Bible reading classes, attending Sunday mass and later on joining a public group of Polish-speaking Young Catholics mark important turning points in young peoples' lives, when they feel that they have become a part of a larger community with shared values.

Becoming a true believer takes time and effort, and involves both collective and individual spiritual activities that are repeatedly referred to in the stories. The collective activities, led by the Catholic priests or the monks of the Polish mission in Sweden, include Bible reading classes, praying, attending Sunday masses, preparations for confirmation and other sacraments. Confession, on the other hand, is an individual ritual through which religious leaders guide the participants in their search for an identity as a believing Catholic. Other activities can be initiated by the young people themselves, although they are financially supported by the Polish mission, such as for instance the regular Young Polish Catholics camps where more experienced attendants welcome the newcomers to the Polish Catholic community. On a personal level, for some of the participants, a relationship with God becomes a life-shaping experience as well as a normal and ordinary part of their everyday life. Faustyna Maj, mentioned above, says:

*I put God in the center of my life because I truly aspire to keep Him in the center. […] He inhabits almost all relationship that I have with other people and I have a personal relationship with God. […] I aspire to live a Christian life: I pray, I meditate, I talk to God in various ways. I consider Him as present in all aspects of my life. […]*.

From Faustyna Maj's perspective, there is no boundary between the sacred and the profane issues in her life. Much in line with McGuire (2008) understanding of religion-as-lived, she describes how most mundane and embodied everyday practices become religious for her. At the same time, upholding the Christian ethos and the desire to live according to its morally defined values can, according to Lundberg (2010, p. 165), be perceived as searching for “depth as opposed to shallowness,” a quest through which young Polish Catholics seek to distinguish themselves from their Swedish peers. Negotiating an ambiguous position that “it is hard to be a Catholic teenager in Sweden” (Drigo, 2017, p. 51), some young people in our study find comfort and strong bonds within the local Polish Catholic community as a way of handling their difficult experiences of not being accepted as they are, and even become more “conservative than their parents” when it comes to faith (Salminen-Karlsson, 2005, p. 57). Other examples from our research indicate that spiritual development helps young Poles to transcend the boundaries of the “ethnic aspects of Catholicism” (Trzebiatowska, 2010, p. 1064) and link them to a wider Catholic community with universal religious values. As Amanda, mentioned above, says:

*Before I thought that I had my own special church, the one I liked, where I could pray. Now, when my faith is stronger and deeper, I am convinced that God is everywhere and I can pray any time and where I choose. It helps me a lot that I can work on my faith anywhere, for instance, at the Catholic festivals for young people or retreats*.

Through faith, Amanda identifies several social spaces for belonging and collective meeting places where God is present, transcending ethnic and geographical boundaries.

### Building New Friendships and Making Sense of Common Migration and Religious Experiences

Arriving in a new country at a young age in a crucial phase in a child's life course is marked by a complexity of experiences triggered by migration but also many choices that the child is eager to make in a society quite different from where they were born. When we meet the Polish participants in this study, most of them are attending university or working. When they came to Sweden they were at the age when socializing outside the family, especially with peers, was extremely important. Schools are usually perceived as “a key site of socialization and acculturation for [migrant] children” (D'Angelo and Ryan, 2011, p. 239). After arrival, primary and secondary schools in Sweden became the social sites where our participants spent most of their time. It does not seem, however, that these were their primary sites for making friends. Some of our participants, who after arrival had to attend the introductory courses in the Swedish language, told us that meeting new friends there “was easy,” because all the others in the group came from different countries and were newcomers to Sweden. However, for others, finding new friends in Sweden was a much more complicated matter. Matilda, whom we introduced earlier, admits:

*When I came to the “Welcome class” I did not have many friends. I did not feel at home in Sweden right away. I attended an elementary school in one of Gothenburg's suburbs, I had problems with being overweight and was bullied because of it in secondary school. Later on, I ended up in a very strange group of “friends” – I didn't like it there*.

During the interview, Matilda came across as a rather shy person, not willing to complain or ask for help. She indicates that after her arrival and for 7 years in Sweden she was struggling to feel comfortable and accepted by her peers. At some point, she was introduced to a group of Polish girls who lived in her neighborhood and attended the catechesis classes in the Polish Catholic church. At first she felt that she was among “her own people.” Later on, when she continued preparing for confirmation, while her “friends” stepped aside Matilda ended her friendship with them. She realized that they were “a gang of troublemakers,” whom she had befriended not because she wanted to, but because everybody else expected her to be “with other Poles.” Similar evidence is presented in Sime and Fox (2015a), who argue that shared ethnicity and language become the first stepping stones to developing friendships post-migration; however cultural differences, stereotypes and class backgrounds grow in importance with time and impact the type and strengths of both inter- and intra-ethnic friendship ties post-migration.

Anna (16, 12 years old on arrival) came to Sweden more recently and appears to be very active in making friends both when she lived in Poland and in Sweden. Despite her enthusiasm and openness to all new contacts in Sweden as well as her agentic attitude in asking adults for help, she admits:

*I talked to the teachers several times and pointed out that no one talks to me at school and I feel lonely. I had to sit alone in the school canteen and eat lunch by myself, I had to do basically all things in school by myself. No one spoke a word to me. The teachers took up this issue once before the whole class, but nothing changed. They did not want to be friends with me*.

As Sime and Fox (2015a, p. 384) underline in their study, for migrant children friendships in a new country are crucial to “overall satisfaction with migratory experience.” For both Anna and Matilda, an experience of feeling excluded by their peers in school lingered for many years afterwards, especially in situations when they had to establish new relationships. In time, both of them and other participants found supportive and caring friends among Swedes and other inter-ethnic networks at high school or at university. Nevertheless, turning to the intra-ethnic friendships was a common strategy to bridge isolation and estrangement, especially during their first year in Sweden.

New friendships post-migration seem to thrive when our participants shared more than a common ethnicity with others. Joining the youth activities of the Polish Catholic Community was not mentioned once as a decisive turning point in the participants' friendship experiences. Again, some of our participants acknowledged that before moving to Sweden they attended compulsory religious education in school and Sunday services at a church back in Poland, the latter together with their parents, but these were described as mainly habitual and non-engaging practices. At times, the participants even expressed that they were in total opposition to the values of the Catholic Church when in Poland. Antoni (25, 18 years old on arrival), for instance, admits his disinterest in traditional religious activities when he was a teenager living in Poland. He arrived in Sweden as almost an adult, after living apart from his parents and his younger brother for 8 years. Before meeting young people through PUKIS (a youth organization for the *Polish Young Catholics*), which has both national and local branches in Sweden, Antoni had a period in his life where he felt lost and lonely. He spent a lot of time reflecting on which direction his life should take, and about his future. It was his mother who advised him to attend a camp organized by the national *Polish Young Catholics* group. Although quite hesitant at the beginning, Antoni decided to go:

*When I look back, I am truly grateful to my mother for persuading me to go to the Catholic camp. It was such fun to meet people of my age, to feel unimpeded in speaking my mother tongue all the time. It turned out that praying was not the only thing we could do all days long; we also had free time, and people I met there had inspiring ideas. […] I felt that I wanted to be with them. It was great to be part of a group where I felt that I really belonged*.

Through the activities within the Polish Catholic community, Antoni was introduced to other young people of his age, many of whom had migrated with their parents from Poland to Sweden. With them, Antoni could not only speak his own language, but also be accepted as he was. He described this as being a life-changing and positive experience, especially when it came to his feelings of finally “fitting in.” Antoni also found his girlfriend at one of the camps of *Polish Young Catholics*. They share the same values, and have the same views and hopes for their future together.

Märta (17, 10 years old on arrival), arrived in Sweden when she was much younger than Antoni. Among her schoolmates in the secondary school there were no children who had come from Poland. In the interview, Märta states that she has many Swedish friends, but it is within the Polish Catholic community that she found her very close, “best friend”—a friend whom she placed in the inner circle of her network map:

*I keep in touch with all of my friends, most of my friends are from Sweden. They understand me - but she [her best friend] understands me better, because she has the same experiences as I have*.

Similar to other young Poles in our study, Märta met her “best friend” through the religious classes and went with her to some of the youth camps arranged by the Polish Catholic Church. Due to their common experience of migration, their strong friendship became an important resource, easing their adaptation and building “a comfort zone in new surroundings” (Holland et al., 2007, p. 102).

Young Poles in the study have also described meeting friends of different ages from among other Polish Catholics on their music festival trips and pilgrimages abroad; friends who did not belong to their local church. Choosing friends who share the Catholic faith is, as Salminen-Karlsson (2005) argues, a way for young people to construct and express their identity. Similar to her findings, some young Poles in our study underlined that socializing with other Catholics of different ages was perceived as much more rewarding, because they could share intimate experiences and receive important advice on navigating their future adult lives as Catholics.

In addition to creating strong personal bonds, being a member of the local *Polish Young Catholics* group provided young people with other important resources. Amanda and Matilda, for instance, were both recruited as group leaders for other young Catholics in the Catholic community. According to Amanda, this assignment deepened her own faith and increased her social competence, in particular when it comes to learning and practicing leadership, responsibility, and social skills:

*My assignment meant that I had to develop a more comprehensive knowledge of our religion, but I also had to practice social relations. I had to start getting to know people, be empathetic and talk to those who experienced problems*.

By investing their time and energy in welcoming and educating other young Catholics, Amanda and Matilda have also performed acts of intergenerational solidarity and loyalty, which, according to McGovern and Devine (2016), are significant affective practices mediating adaptations in a new country for themselves but also other migrant young people within the Polish Catholic community.

The efforts expended by more experienced young Catholics in inspiring and guiding others seem to be very much appreciated. Vera (18, 6 years on arrival), is one of those participants who admitted that her faith and attitude to religious practice had swung back and forth over the years. At times, it helped her to take her mind off emotional distress caused by difficult relationships with her brother and her father. At other times, she could identify with other young Poles, who were reluctant to take part in Catholic catechesis classes. Vera explains:

*Most of the children who met for the catechesis classes and the like, they were going […]: ah, I can't stand it here, I don't want to be here. When they meet the group leaders, who are really devoted and for whom faith is a free choice, they realize that there is like an energy they get from them, an energy they can also pass on to others*.

Needless to say, during the time of ongoing pandemic, when masses in church, praying and confession, etc., have been more or less put on hold or transferred online, young Poles are experiencing an even stronger need for empathy and friendly talk. In order to not feel alone and keep their friendships with other young Catholics, some participants in the study displayed significant agency by creating smaller WhatsApp and Facebook groups for praying and daily communications.

For those who upon arrival in Sweden did not feel welcomed by their peers in primary and secondary schools, finding reliable friends within the local Polish Catholic community helped to rebuild their self-esteem. Following McGovern and Devine's (2016) argument, through these supportive relationships, young Polish migrants in our study felt that they were recognized and accepted by others, which could infuse them with a confidence that they will be included and succeed in building caring relationships even outside the Polish Catholic community.

At the same time, within this social arena several young people were reassured not only about their worth and faith, but also about their Polish heritage. Young people's catechesis readings, preparations for confirmation, camps and confessions are all conducted in the Polish language. Polish is, for the most part the only language that the participants speak within their families, but also with their “best friends,” whom they meet in the Polish community. Keeping their own language alive reaffirms young migrants' sense of strong continuity with their home country, even if they spent most of their formative years in Sweden. Jan (21, 13 years old on arrival) tells us with some affection how proud he is to be fluent in Polish:

*When I visit Poland, I feel so happy that I can understand everything that people say. I can go to a shop and ask [a happy smile]: I would like to buy a bun! Oh. And I can say it in Polish. It feels so amusing that they understand me. I can also go to a doctor and speak Polish, I can speak only Polish, a language that I […] [I: that you love?] Yes, indeed*.

Jan has exclusively Polish friends in Sweden, many of whom he met through the Polish Catholic community. He speaks Polish with his parents and younger siblings, consumes books and TV programmes in his native language, and occasionally uses Polish in his job. What he seems to be especially proud of is that his Polishness is acknowledged back in Poland. Jan and some other participants in the study, clearly express what Slany and Strzemecka (2016, p. 19) categorize as a univalent type of national identity, meaning “a special attachment to the Polish culture,” as if they are living in “little Poland” abroad.

For Antoni, whom we introduced above, the group of *Polish Young Catholics* also became a vital part of his life, with a huge impact on his ideas and thoughts. He states that religion is an important part of the Polish way of life, where traditional family values are central. When Antoni first came to Sweden, he was critical of these traditional values, and often criticized the way his parents lived—and the way they wanted him to live. Due to his strong relationship with one of the monks and engagement within the *Polish Young Catholics*, he changed his views on faith, which greatly strengthened his relationship with his parents and the extended family back in Poland.

One of our participants, Antonina (29, 16 years old on arrival), belongs to another, non-Catholic Christian congregation in Sweden. She described how upon her arrival she could continue to follow her religion but within a Swedish-speaking religious community. Antonina underlines this point when we asked her about her friends:

*Right now I am in a phase of my life when I don't have much contact with those Swedish friends whom I met when I started to attend this church 7 years ago. […] If you would have asked me the same question 7 years ago, then among my friends and important people I would definitely have included a lot of Swedes*.

Antonina's story illustrates how another multi-ethnic religious congregation has been transformed into a mono-ethnic community due to the increasing numbers of arrivals of the Polish laity in Sweden. Her example is similar to Jan's in a way, as she also admits that she basically lives in “little Poland” abroad. At the same time, her story reminds us that while religious practice can facilitate young people's networks within the host society, it can just as well reverse their adaptation by reducing their engagement with the non-Polish community.

### Polish Catholic Priests' Guidance in Family Matters and Future Life Projects

The significance of specific religious actors within the Polish Catholic community emerged clearly in the young Poles' narratives. Almost all of the young Poles we have interviewed so far identify one or two priests or monks as the most important people in their personal networks. Four out of 11 place a priest or a monk in the inner circle of their individual network maps, indicating that, similar to parents, siblings and closest friends, priests and monks play a pivotal role in their lives post-migration. We view the care and mentoring work that these specific religious actors perform in young people's lives as following Reynaert's (2014) and McGovern and Devine's (2016) conceptualization; namely that it is through the investment of time, attentiveness and engagement that these specific Catholic priests and monks became “significant others” to the young Poles in our study. It is important to note that several of our participants distinguished between “good” and “bad” priests. For instance, Amanda, Matilda and Faustyna Maj admit that their engagements within the Polish Catholic community are highly influenced by the great personal devotion, and the positive and accepting attitude of the priests and monks working in the community. These are unanimously described as open, interested, non-judging and warm representatives of the Catholic Church. Anna, whom we introduced above, recalls that she was initially reluctant to follow the tradition and to take the holy sacrament of confirmation. Attending the catechesis classes at the time of interview, she got to know another priest through confession and brief conversation, and gradually changed her position:

*He was always so kind, he always cared about me and showed it, by saying: Yes, you are a very important person. It was […] I must say […] he was just such a good person who helped me to find a way back to myself in a sense*.

By embracing this attitude, the monks and priests have come to play a crucial role as mentors and guides in the lives of these young people, exercising what Reynaert (2014) categorizes as non-hierarchical pastoral power. Priests' and monks' genuine ways of “practicing what they preach” inspired the participants to listen to their own spiritual voices. Reflecting upon how her spiritual clarity took shape, Faustyna Maj explains:

*It was thanks to these people who, in my opinion, are called to be priests. […] They follow their faith and they practice what they preach. In some sense, what they do is radical, it is honest. And I know that this is the way it should be. That is why I admit that it is due to these people I got to know God, faith, Jesus and what the church should be like*.

It is important to underline is that the local Polish Congregation in Gothenburg is led by the priests and monks of the Capuchin Order, living in a monastery. As one of the participants clarified in an informal conversation, different Catholic orders have “different charisma:” some follow a call to spread joy and happiness, and others emphasize spiritual learning and charity. The key mission of the Capuchin Brothers is to preach and to serve as confessors, especially when it comes to children and young people (Capuchin Order in Sweden).

What we would like to highlight is that the priests and monks, besides providing religious education leading to confirmation, also provide different kinds of social support and guidance in other life-related matters. One issue of central importance is helping young Poles to resolve family conflicts and to overcome the ruptures in family relationships caused by migration. Most of our participants had lived at a distance from one or both of their parents for between 2 and 7 years before the family reunited in Sweden. As observed in other studies on European migrant families (Sime and Fox, 2015a; Moskal and Tyrrell, 2016; Tyrell et al., 2019), children usually lack full agency in migration decisions and have to cope with multiple separations from extended family, friends and even siblings. Step-migration of this kind is often accompanied by feelings of frustration and anger, causing tensions and emotional distress long after young migrants join their parent(s) in Sweden and try to learn how “to live together again.” Reuniting in Sweden often leads to changing family roles due to the young people becoming young adults, wanting to decide for themselves. The religious leaders whom young Poles ask for advice in these matters reconcile these tensions by involving the entire family.

Monica, for instance, explains that the priest provides support for the whole family and when he visits them every Christmas he blesses everyone in the family as well as sharing a good and well-prepared meal with them in their family home. For her part, during her individual confessions with the priest, Monica expresses how she cares for her family by seeking advice on conflict resolution:

*[.] most of [the confessional talks] is usually about my relationship with my parents and especially with my mother, [on something] we don't agree on […], the reason for our dispute and how to deal with it. Sometimes you need time to understand how things really are. With time I can get a perspective on what the priest has actually told me [during my confession]. Then I feel I might be able to start talking about it [with my mom]*.

Monica and other young people tell us that the specific conflicts in the parent-child relationship generally require several steps of family guidance. In those cases, the priest, as Monica describes, gives separate guidance to each family member and later on, if they prefer, they can share the individual advice they received with each other. The way the priest supports Monica's family is echoed in other stories as well; for instance in the case of Antoni. As indicated above, his confessor helped him to embrace Catholic religious values and the traditional Polish way of life. In this way, Antoni obtained a better understanding of his parents' decisions and views, and felt closer to them. Guiding young people in their actions as well as feelings in relation to their families, the priests perform care work, which according to McGovern and Devine (2016) is important for the youngsters' well-being, but also for sustaining family bonds and creating mutual respect across generations.

We attribute the centrality of the specific religious actors in young Poles' lives as due to their engagement and empathy, which makes the young people feel that they are “understood and taken seriously by others.” In this way they bestow on young people continuous guidance and even practical support in building their identities and important social networks post-migration. Several participants emphasized the value of personal discussions with a priest on how to live one's life, in addition to religious norms and morals. Wanda, mentioned above, reflects:

*Yes, I can get some guidance for my life in the Catholic church's catechesis and so on. […] But it is much more important that I can talk through these things person to person, and not only being told what the church and the Bible say about it*.

*I: No, I see*.

*They [the priests] usually explained that there are two sides of the coin. One is what the church says about the issue, but another is how you choose to act on the issue*.

*I: Right, so you had an option to take your own decisions?*

*Of course, in the end it is always one's own decision that matters*.

Similar to Wanda, Monica, Amanda and Vera also emphasized that during confession or little conversations at the camps, specific priests and monks, in line with Reynaert's (2014) understanding of pastoral power, provided young people with questions to reflect on, instead of ready answers to how to behave. Following McGuire's (2008) understanding of religion as a lived experience, Wanda indicates that the individual talks with a priest on issues that were both sacred and mundane made the most sense to her in her understanding of what was important in her life.

Other participants admitted that they discussed their decisions with a monk or a priest when considering choices about their future education. On several occasions, young Poles could count on the priests' and monks' practical support, for instance by connecting them to resources such as other community members, getting a ride to another town or being helped into a work placement.

Matilda, whose story we referred to in the beginning, describes the religious actors within the community as people who she trusts and whose help she can count on at any time:

*[.] if I, for example, have a problem [ …] in any relationship or a moral problem with someone or, if I have a problem with myself or whatever and it is something that feels difficult, or I feel I have to talk [about it] to someone, it feels like [they are]… yes, it's almost like my other family. [laugh]*.

Matilda's description of the Polish Catholic community and especially the religious leaders within it as “another family,” echoes other participants' acknowledgment of the community's essential role as a complement or even as an alternative to the formal social security system in Sweden. In line with Reynaert (2014), the priests' extensive pastoral care serves as an extra buffer for young people, especially when they might lack support from their families or friends.

In a similar way, Antoni describes how he highly values his relationship with one of the monks, being both a religious authority to him, to whom he confesses, but also a knowledgeable professional, who treats Antoni with respect, almost like a good friend:

*[.] He is not just a person who knows the Bible [he is] more like a psychologist to me [.] I usually go to confess to him. I can go and talk to him quite honestly because he knows all about me anyway. He never judges me or anything [.], the only thing he does is listen and listen and listen to me and then, at times, he interjects some hints. He never forces me to do anything [.] he listens, he is a very good listener, and then, he just says what he would have done or what his experience is with a certain matter. Then he usually says that the final decision always lies with me. So he is like a good friend of mine and at the same time like a psychologist, though completely free, because I … I do not have to pay a single cent*.

The authority of the priest who Antoni cherishes so dearly lies not in forcing a young man to act in a certain way, but in allowing room for an exchange of thoughts and doubts. In Antoni's and other young Pole's stories, the priests emerge as trustworthy mentors, active and caring listeners, encouraging young people's agency to express themselves and take their own decisions.

## Concluding Thoughts

This article is based on a small qualitative study and the results we present here cannot be generalized to the whole population of young Poles, arriving in Sweden since 2004 as a result of family migration. Our analysis has nevertheless offered some insights into the significant role that the faith community can play for the young migrants' identity construction, and in building meaningful social relationships post-migration. To begin with, we reviewed the previous research on young people's mobility, identity and religion, which are usually framed as separate research fields. Adopting relational and affective perspectives (McGovern and Devine, 2016) on the young Poles' post-migration experiences revealed that besides family and schooling, a great many of the adaptation and identity processes took place within the realm of the Polish Catholic community in Sweden.

Several of the young people identify their relationships with God, the priests, and friendships generated within the Polish Catholic community as positive turning points in their lives post-migration. These results should be seen through the lens of other observations from the current study, namely that many of our participants experienced difficulties while socializing and making new friends in Sweden, especially during the first year post-migration. Several of them talked about being victims of bullying and discrimination in school. Even those young people, who had Swedish-born friends, admitted that their native Swedish peers lacked the experience of migration and loss caused by leaving close friends and family members in Poland.

Adams (2009) call to listen to the spiritual voices of children is applicable here, pointing to the fact that children's and young people's quest for spirituality and faith was recognized within the Polish Catholic community and its institutional structures, and by specific religious leaders, who cared about young people's well-being as human beings. The community's priests and monks, in the spirit of Reynaert's (2014) notion of pastoral power, offered guidance for young people to help them cope with a sense of insecurity, affirm their religious faith, and to independently find their own answers to the complex questions in life.

We have argued in this article that socializing within the Polish Catholic (and other religious) communities can be better understood through the lens of the lived-religion perspective (McGuire, 2008), blurring the boundaries between the sacred and mundane practices in the young people's lives. Along with spiritual development, they formed long-lasting friendships and even some conjugal relationships within the community. Similar experiences of migration in childhood or youth seemed to produce a longer-term impact on the participants' lives and significant social relationships. Especially for those young Poles who felt excluded by their peers and not welcome, the Polish Catholic community has become an important space of solidarity and recognition between peers who have common experiences in struggling with starting a life in a new country.

The article shows how weekly activities within the church, youth camps for young Catholics and other occasions within the community are also perceived as spaces of belonging because the participants can speak their native language with people who understand their situation. Many of the young people who receive caring support and spiritual guidance from the priests and friends within the Polish Catholic community tend to also give back to the community by taking on leadership roles in youth groups, supporting their peers as spiritual guides together with the priests, or by serving coffee together with other youngsters after Sunday masses. In this way, the participants in the study become themselves active agents in the Polish Catholic community, promoting shared religious values, solidarity, and mutual support.

In wider terms, this article also argues that the Polish Catholic community facilitates family sustainability post-migration and represents a space of social security and trust across generations, a web of social networks which young Poles can count on in a situation of crisis. What merits further investigation is the fact that while practicing religion and building deep social relationships within the Polish congregations shapes the young migrants' feelings of belonging and inclusion, it takes place primarily within the limits of their own ethnic community. This finding raises a question about the wider implications of the mono-ethnic relational practices for the young Poles' lives within the increasingly ethnically heterogeneous Swedish society.

## Data Availability Statement

The datasets presented in this article are not readily available because the data cannot be shared with anyone else but the researchers on the project. Requests to access the datasets should be directed to oksana.shmulyar@socav.gu.se.

## Ethics Statement

The studies involving human participants were reviewed and approved by Swedish Ethical Review authority [Dnr: 2019-02504].

## Author Contributions

This article has been written in a close collaboration between all authors. The empirical material has been collected by OSG and CM. The material has been analyzed by all three researchers. The article was drafted by the OSG and CM with initial contributions from all three authors. The article has been finalized by the OSG.

## Conflict of Interest

The authors declare that the research was conducted in the absence of any commercial or financial relationships that could be construed as a potential conflict of interest.

## Footnotes

^1^The project *Transnational childhoods: building of significant relationships among Polish and Romanian migrant children after reunification with parents in Sweden* [2018-00369], led by professor. Ingrid Höjer, received funding from the Swedish Research Council for Health, Working Life and Welfare (FORTE, 2019–2021).
